# Frequency of Exfoliation Syndrome Among Patients Attending an Ophthalmology Outpatient Clinic in the Northeastern Black Sea Coast of Turkey

**DOI:** 10.3390/healthcare14070877

**Published:** 2026-03-28

**Authors:** Demet Yabanoglu, Ayse Tulay Bagci Bosi, Naciye Kabatas, Emrah Utku Kabatas, Murat Irkec

**Affiliations:** 1 Department of Ophthalmology, Faculty of Medicine, Hacettepe University, Ankara 06100, Turkey; muratirkec@gmail.com; 2 Department of Ophthalmology, Rize State Hospital, Rize 53100, Turkey; aktasnaciye@yahoo.com; 3 Institute of Public Health, Faculty of Medicine, Hacettepe University, Ankara 06100, Turkey; tulaybagcibosi@gmail.com; 4 Department of Ophthalmology, Dunyagoz Hospital, Izmir 35220, Turkey; 5 Department of Ophthalmology, Provincial Health Directorate of Izmir City Hospital, Izmir 35540, Turkey; dremrahutku@yahoo.com; 6 Department of Ophthalmology, Ishakoglu Cayeli State Hospital, Rize 53200, Turkey

**Keywords:** cataract, comorbidity, cross-sectional studies, epidemiology, glaucoma, pseudoexfoliation syndrome

## Abstract

**Highlights:**

**What are the main findings?**

- In a hospital-based surveillance of patients aged ≥40 years (n: 938) attending a regional ophthalmology outpatient clinic, exfoliation syndrome was common, with an overall hospital-based frequency of 20.8%.
- Exfoliation syndrome showed strong age dependence and was associated with glaucoma and hypertension in this care-seeking population.

**What are the implications of the main findings?**

- Hospital-based exfoliation syndrome frequency data can inform regional service planning for glaucoma surveillance and cataract care, but should not be interpreted as population prevalence.
- Routine slit-lamp screening for exfoliation syndrome in older clinic patients may improve risk stratification for glaucoma surveillance and perioperative cataract management in regional referral centers.

**Abstract:**

**Background:** Exfoliation syndrome (XFS) is a common age-related disorder and a major risk factor for glaucoma and cataract. This study describes the hospital-based frequency and clinical associations of XFS among patients aged 40 years and older attending a regional ophthalmology outpatient clinic in the Northeastern Black Sea coastal region of Turkey. **Methods:** This cross-sectional observational study included 938 eligible participants aged ≥40 years with registered birth records and continuous residence within the defined catchment area who underwent a comprehensive ophthalmological examination. XFS was defined by characteristic exfoliative material at the pupillary margin and/or on the anterior lens capsule (phakic eyes) or capsular bag/IOL complex (pseudophakic eyes), with pupillary assessment before and after pharmacologic dilation. Systemic comorbidities were extracted from national medical records. Multivariable logistic regression adjusted for age and gender. **Results:** XFS was diagnosed in 20.8% (195/938; 95% CI: 18.2–23.5%). The XFS-positive (XFS+) group was older than the XFS-negative (XFS−) group (71.76 ± 0.61 vs. 63.25 ± 0.40 years; *p* < 0.001). Hypertension was more common in XFS+ participants (57.4% vs. 45.6%; *p*: 0.002) and remained associated after adjustment (OR: 1.49; 95% CI: 1.05–2.11; *p*: 0.024). Glaucoma was more frequent in XFS+ participants (23.6% vs. 14.9%; *p*: 0.005); it remained associated after adjustment (OR: 2.00; CI: 1.31–3.05; *p*: 0.001). **Conclusions:** In this hospital-based surveillance, approximately one in five clinic attendees aged ≥40 years had XFS. Findings should not be extrapolated to population prevalence; population-based studies are required to estimate regional prevalence accurately. Nonetheless, these data highlight a substantial clinical burden of XFS in a regional care-seeking population and support vigilant glaucoma surveillance in affected patients.

## 1. Introduction

Since the seminal description of exfoliation syndrome (XFS) by John G. Lindberg in 1917, numerous studies have investigated its clinical characteristics and associations with both ocular and systemic conditions [1,2,3,4,5]. Recognizing XFS is of major clinical importance, as the condition is associated with serious vision-threatening ocular complications, most notably glaucoma, as well as a range of systemic manifestations that may carry prognostic significance [6,7,8,9,10,11].

The occurrence and clinical presentation of XFS have been reported to vary widely across different settings, attributable to differences in diagnostic approaches, genetic background, and geographic or environmental factors [5,12,13,14]. International studies illustrate marked heterogeneity in reported rates across regions, with reports ranging from very low levels in some East Asian population-based surveys to substantially higher proportions in Northern European studies, underscoring the multifactorial nature of the syndrome and highlighting the need to interpret findings within the context of study design and setting [15].

Although Lindberg originally used the term “exfoliation syndrome”, subsequent authors have proposed alternative nomenclature to describe the same pathological process [16]. The prefix “pseudo” was introduced to distinguish this condition from true exfoliation syndrome, a rare delamination of the anterior lens capsule classically described in individuals exposed to intense heat/infrared radiation [1]. Given the rarity of true exfoliation, the term XFS has remained widely used in the literature; in the present study, the term XFS is used in accordance with this convention.

The Northeastern Black Sea coastal region, including the province of Rize where the present study was conducted, has several geographic and demographic characteristics that distinguish it from other regions of Turkey where XFS data have been reported [17,18]. Unlike many previously studied areas of the country characterized by continental or Mediterranean climates, this region has a humid oceanic climate with high levels of precipitation, persistent cloud cover, and relatively stable temperatures throughout the year [19,20]. Previous epidemiological studies have reported marked geographic variation in the frequency of XFS, with particularly high frequencies observed in Northern European populations compared with Mediterranean and other regions [15]. These observations have led to the hypothesis that environmental factors, including latitude and climatic conditions, may contribute to regional differences in the occurrence of XFS. Therefore, the distinct geographic and climatic characteristics of this region may be relevant when interpreting regional variations in the frequency and clinical characteristics of XFS [21,22].

Epidemiological data on XFS in the Northeastern Black Sea region of Turkey remain limited. To the best of our knowledge, no previous study in this region has systematically evaluated individuals diagnosed with XFS through comprehensive ophthalmological examination. Although previous studies from Turkey have investigated the frequency and clinical features of XFS in various settings, comprehensive evaluations based on consecutive patients presenting to a regional ophthalmology clinic remain scarce [17,18,23]. By screening all patients consecutively attending a regional referral center, this study provides hospital-based surveillance data that may offer a more representative insight into the occurrence of XFS in the Northeastern Black Sea population.

The diagnosis of XFS requires slit-lamp biomicroscopic examination and appropriate ophthalmic equipment, making population-based field screening difficult to implement under the current healthcare conditions in Turkey. Therefore, the systematic evaluation of patients attending a regional referral ophthalmology clinic may provide valuable epidemiological insight into the occurrence of XFS within the community served by the institution.

Furthermore, the practical challenges of population-based screening and the relatively low level of disease awareness among individuals may contribute to delays in diagnosis and treatment. Given the well-established association between XFS and an increased risk of glaucoma, early recognition of the disease remains clinically important [4,5].

In this context, this study aimed to investigate the frequency and clinical associations of XFS among patients presenting to a regional ophthalmology clinic in the Northeastern Black Sea coast of Turkey. The primary objective of this hospital-based surveillance study was to estimate the frequency of XFS among patients undergoing routine ophthalmic examination at this clinic. The secondary objective was to explore ocular and systemic associations of XFS within this clinical population. By describing these associations, the study sought to increase clinical awareness among healthcare providers and provide additional insight into the clinical characteristics of XFS in this region.

## 2. Materials and Methods

This hospital-based cross-sectional observational study was conducted in accordance with the Declaration of Helsinki and was approved by the Ethics Committee for Clinical Studies of Rize University, Faculty of Medicine (approval number: 2012/37-43). Written informed consent was obtained from all participants.

**Figure 1:**
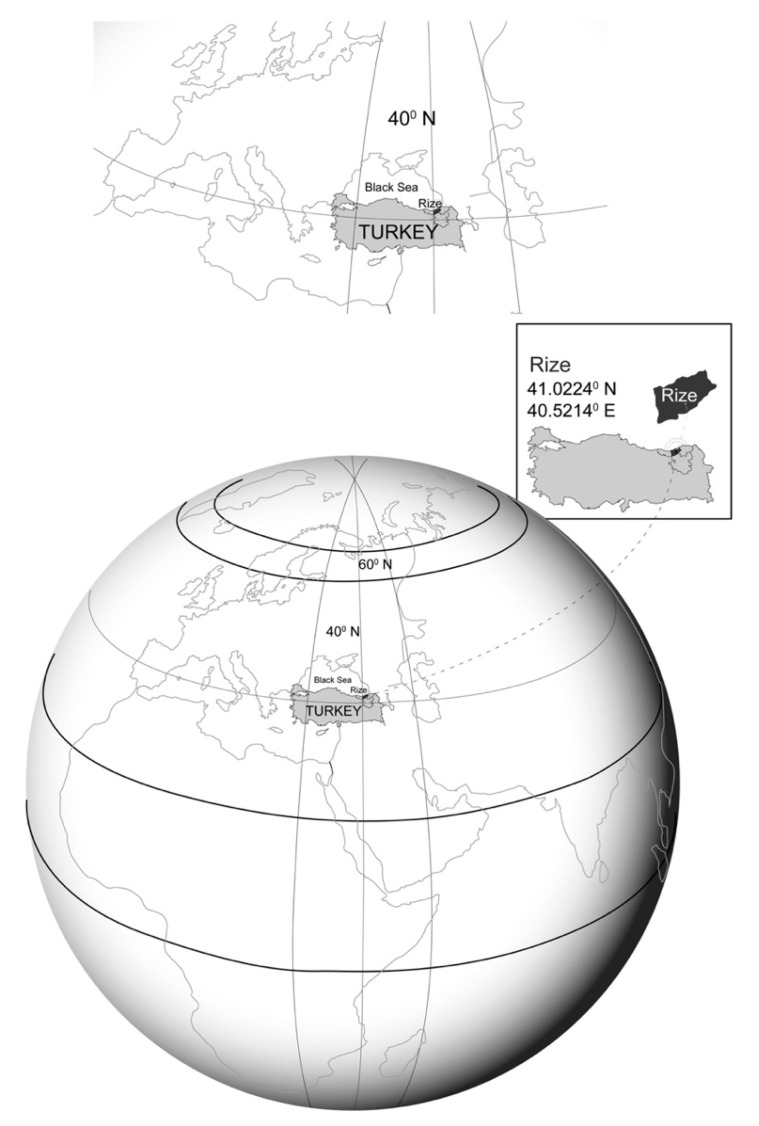
Annotated map showing the geographic boundaries of the study area within the Northeastern Black Sea coastal region of Turkey.

The study was conducted at the State Hospital of Rize, located at 41° N latitude on the Northeastern Black Sea coast of Turkey. The catchment area comprised the provincial center of Rize and its surrounding coastal districts, which defined the geographic boundaries for inclusion (Figure 1).

**Figure 2:**
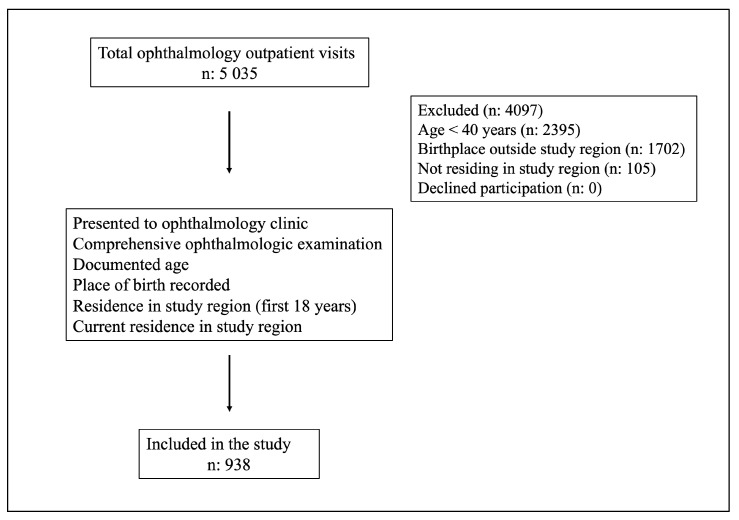
Flow diagram of the participants in the study (n: 938).

During the 12-month study period, a total of 5035 outpatient visits were recorded at the ophthalmology outpatient clinic. All consecutive patients aged ≥40 years who were born in the study region, had continuous residence in the region, and met the predefined inclusion criteria during this period were evaluated according to the comprehensive ophthalmological examination protocol. No exclusion criteria were applied for patients with a prior diagnosis of glaucoma or a history of ocular surgery. No sampling procedure was applied; therefore, the study represents a consecutive hospital-based surveillance. After applying the predefined eligibility criteria, 938 participants were included in the study as shown in Figure 2.

**Figure 3:**
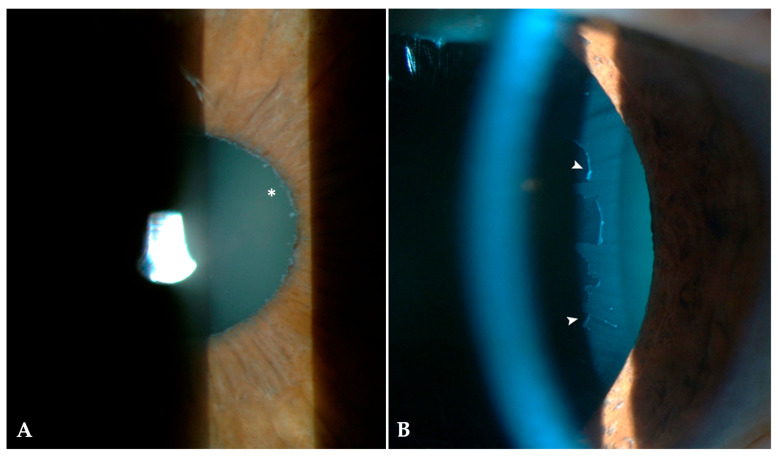
Slit-lamp Features of XFS. Slit-lamp photograph showing whitish fibrillar deposits characteristic of XFS. (**A**) Deposits along the pupillary margin in a non-dilated pupil (asterisk). (**B**) Deposits on the anterior lens capsule in pharmacologically dilated pupil (arrows).

All participants underwent a comprehensive ophthalmological examination, including best-corrected visual acuity assessment (BCVA), measurement of intraocular pressure (IOP) and central corneal thickness (CCT) using the same non-contact tonometer with integrated pachymetry (TX-20P; Canon Medical Systems, Tokyo, Japan), calibrated according to the manufacturer’s recommendations, slit-lamp biomicroscopy (BQ 900, Haag-Streit AG, Koeniz, Switzerland) with pharmacologic pupillary dilation (tropicamide 1% ophthalmic solution, Tropamid^®^ Bilim Ilac, Istanbul, Turkey), fundoscopic examination, and evaluation of the cup-to-disc ratio (c/d). Lens status was recorded for each eye as phakic or pseudophakic. Phakic lenses were further categorized as nuclear, cortical, anterior subcapsular, or mature cataract. The pupillary margin was assessed before and after pharmacologic dilation. The diagnosis of XFS was based on slit-lamp biomicroscopy with detection of characteristic XFM on the pupillary margin (Figure 3A), and/or the anterior lens capsule following pharmacologic pupillary dilation (Figure 3B) in phakic eyes. In pseudophakic eyes, XFS was diagnosed based on XFM on the capsulorhexis edge/residual anterior capsule or the IOL-capsular bag complex when visible. In cases where these structures could not be adequately visualized, gonioscopy was performed to aid in the detection of XFM. Participants were categorized as XFS-positive (XFS+) or XFS-negative (XFS−) based on these findings. In XFS+, laterality was recorded as unilateral or bilateral. Glaucoma diagnosis was established according to the criteria of the European Glaucoma Society Guidelines and was determined by three experienced ophthalmologists (DY, NK, EK) to ensure diagnostic consistency [24]. Borderline cases were periodically cross-checked among the same ophthalmologists approximately every 25–30 patients to maintain interobserver consistency.

Based on their XFS status, the participants were categorized into four groups as follows: Bilateral XFS+, Right eye XFS+, Left eye XFS+, and XFS−. Ophthalmological findings were compared across three categories. In the first analysis (group analysis), XFS+ and XFS− groups with the same laterality were compared. In the second analysis (corresponding analysis), corresponding eyes within the subgroups were compared.

National medical records were reviewed to identify systemic comorbidities, including hypertension (HT), coronary artery disease (CAD), diabetes mellitus (DM), cerebrovascular disease (CVD), and hyperlipidemia (HL). Comorbidities were defined using documented diagnoses in the national electronic health record and/or active chronic medication prescriptions recorded prior to or at the index visit.

### Statistical Analysis

After data validation, categorical variables were summarized as counts and percentages, and continuous variables as means ± standard errors ($\;\bar{X}$ ± SE). Normality was assessed using the Kolmogorov–Smirnov test. Group comparisons were performed using ANOVA and Student’s *t*-test for continuous variables, and the Chi-square or Fisher’s exact test for categorical variables, as appropriate. Statistically significant cut-off is 1-α: 0.05. In the multivariable logistic regression analysis, XFS positivity was considered the dependent (outcome) variable. All clinically relevant variables evaluated in the study were initially examined for their association with XFS and were considered for inclusion in the model. Interaction and correlation among independent variables were assessed using correlation analysis, and variables demonstrating relevant associations were entered into the multivariable model. Potential correlations among independent variables were examined, and no significant collinearity was found. Age and gender were retained in the model for adjustment because of their clinical relevance and statistical significance (*p* < 0.05). All analyses were performed using IBM SPSS Statistics version 23.0 (IBM Corp., Armonk, NY, USA). No a priori sample size calculation was performed because the study included consecutive eligible clinic attendees; the precision of the primary frequency estimate is reported with a 95% confidence interval (exact binomial).

## 3. Results

In total, 938 individuals were included; XFS was identified in 20.8% (195/938; 95% CI: 18.2–23.5%). Over the 12-month period, XFS was detected in approximately one out of every five individuals who presented to the ophthalmology outpatient clinic. The distribution of participants by gender and age group is shown in Table 1 and Table 2. The mean age of the XFS+ group was 71.76 ± 0.61 years, compared with 63.25 ± 0.40 years in the XFS− group; this difference was statistically significant (*p* < 0.001). Independent of age, the likelihood of XFS occurring in men is 1.865 times higher than in women, and this difference is significant (Table 2).

The frequency of HT was significantly higher in the XFS+ group (57.4%) than in the XFS− group (45.6%) (*p*: 0.002). Glaucoma was more frequent in the XFS+ group (23.6%) than in the XFS− group (14.9%) (*p*: 0.005). Age- and gender- adjusted results are presented in Table 3. Among participants with glaucoma, a subset (n: 157) was receiving topical anti-glaucoma medication at the time of examination, potentially lowering measured IOP values and influencing intergroup comparisons. Since a substantial proportion of participants (n: 157, 16.7%) were receiving antiglaucoma therapy, a further analysis was conducted after excluding treated glaucoma patients. After this exclusion IOP was compared between XFS+ and XFS− groups, and no statistically significant difference was observed.

Nuclear cataract was the most common lens status in both eyes (right eye: 692/938, 73.8%; left eye: 697/938, 74.3%), followed by cortical cataract (right eye: 194/938, 20.7%; left eye: 193/938, 20.6%). Anterior subcapsular cataract and mature cataract were uncommon (each <1% in both eyes). Pseudophakia was observed in a small minority of eyes (right eye: 36/938, 3.8%; left eye: 33/938, 3.5%).

Group analysis is presented in Table 4. In the XFS+ group, the mean BCVA and CCT were significantly lower in both eyes compared to the XFS− group (*p* < 0.050).

Corresponding analysis is given in Table 5. In unilateral XFS+ cases, the mean IOP of the contralateral eye was significantly lower compared to the XFS+ eye (*p* < 0.05). In Left eye XFS+ group, the mean c/d of the left eye was significantly higher than that of the right eye (respectively, 0.21 ± 0.32, 0.15 ± 0.16; *p*: 0.010).

In the subgroup analysis (Table 6), the XFS− group exhibited significantly in both eyes. The higher IOP values were mainly attributable to the right eyes of patients with Right XFS+ group, which showed significantly higher IOP compared with other groups.

## 4. Discussion

In this cross-sectional hospital-based surveillance study, XFS was identified in a substantial proportion of patients aged 40 years and older presenting to a regional referral ophthalmology outpatient clinic in the Northeastern Black Sea coastal region of Turkey. The observed proportion should be interpreted within the context of the study design, as hospital-based samples inherently differ from population-based cohorts [25]. Accordingly, direct numerical comparisons with previously published national or international studies are not appropriate, given the considerable heterogeneity in study populations, sampling strategies, and diagnostic methodologies reported in the literature.

It is well recognized that reported figures for XFS vary widely across studies, reflecting differences in research design, clinical examination protocols, and the characteristics of the populations under evaluation [26]. Even among hospital-based investigations conducted within the same country, methodological differences—such as the routine use of pharmacologic pupillary dilation—may substantially influence detection rates [18]. In this regard, the consistent application of a standardized dilated slit-lamp examination in the present study was intended to ensure diagnostic uniformity rather than to confer a methodological advantage.

Geographic and environmental factors have long been discussed as potential contributors to regional variability in XFS [27]. Northern European regions have frequently been cited as areas with a high clinical burden of XFS, while reports from other parts of the world demonstrate marked heterogeneity [28]. The Northeastern Black Sea coastal region represents a geographically distinct area characterized by a humid oceanic climate, high precipitation, and persistent cloud cover [19,20]. Although environmental variables such as ultraviolet exposure, humidity, and temperature have been proposed as potential modifiers in XFS pathogenesis, the present study did not directly assess these factors. Consequently, any discussion of environmental influences should be considered exploratory and hypothesis-generating rather than causal.

Rize State Hospital serves as the primary public ophthalmic care provider for the defined catchment area, receiving referrals from the city center and surrounding coastal districts. Outpatient visits to this center largely reflect regional care-seeking patterns among adults, particularly those aged 40 years and older. Within this context, hospital-based evaluations may provide meaningful insight into the clinical burden and presentation of XFS among patients seeking ophthalmic care in this geographically defined region, without implying population-level estimates.

Demographic analyses revealed that although female patients constituted a larger proportion of clinic attendees, XFS was more frequently observed among male patients. This finding is consistent with several previous reports suggesting a higher susceptibility or more pronounced clinical expression of XFS in men [29]. Advancing age was also strongly associated with the presence of XFS, in line with established evidence indicating that age-related structural and biochemical changes in ocular tissues play a central role in the pathogenesis of the syndrome [3].

The association between XFS and glaucoma observed in this study underscores the clinical importance of recognizing XFS in routine ophthalmic practice. The frequency of glaucoma was significantly higher among patients with XFS compared with those without XFS, and this association remains significant after adjustment for age and gender (Table 3). Considering that a substantial proportion of glaucoma patients receiving antiglaucoma therapy, further analyses were performed to evaluate the potential confounding effect of treatment on IOP comparisons. These analyses showed no significant difference in mean IOP values between XFS+ and XFS− groups, suggesting that a topical antiglaucoma therapy did not substantially influence the comparison of IOP between the groups. However, this finding should be interpreted with caution, as important clinical parameters such as glaucoma duration and treatment regimens were not evaluated in this analysis. Overall, these findings support the importance of careful and ongoing glaucoma surveillance in individuals with XFS, particularly in high volume referral ophthalmic centers.

XFS has been reported to be associated with several systemic conditions, particularly cardiovascular or vascular disorders [15]. Although an association between XFS and HT was observed, the cross-sectional design of the study precludes any inference regarding causality. Longitudinal cohort studies with standardized cardiovascular assessments would be required to clarify the temporal and mechanistic relationships between XFS and systemic vascular conditions.

Ophthalmological examination findings demonstrated that the presence of XFM, irrespective of laterality, was associated with reduced BCVA. This observation is consistent with previous studies reporting an increased prevalence of nuclear lens changes in eyes affected by XFS [30]. In addition, unilateral XFS was associated with higher IOP values, emphasizing the importance of close monitoring even in cases without bilateral involvement. The lower CCT values observed in patients with bilateral XFS further highlight the potential for underestimation of true IOP in routine clinical settings, reinforcing the need to consider corneal biomechanics when managing these patients.

In pseudophakic eyes, the detection of exfoliative material may be more challenging because the anterior lens capsule is partially removed during cataract surgery, and the capsulorhexis margin or the intraocular lens–capsular bag complex may limit the visualization of residual exfoliative deposits. Previous studies have reported that the diagnostic sensitivity for pseudoexfoliation may decrease after cataract extraction, as some of the characteristic findings on the anterior lens capsule become less visible following surgery [31,32]. This limitation may lead to potential under detection of XFS in operated eyes. To minimize this possibility, suspected cases were carefully evaluated by reviewing available preoperative medical records when accessible, and gonioscopy was performed in cases where slit-lamp findings were inconclusive.

Several methodological considerations should be acknowledged. The hospital-based design may have influenced the observed clinical profile, as individuals seeking ophthalmic care are more likely to present with ocular disease than the general population. Systemic conditions were verified through national medical records. Although ophthalmologic examinations were performed by multiple ophthalmologists; a standardized protocol including pupillary assessment before and after pharmacologic dilation was applied. To maintain interobserver consistency, borderline cases were periodically cross-checked jointly by the same ophthalmologists approximately every 25–30 patients, in an effort to minimize potential discrepancies.

Another consideration is that patients with a prior diagnosis of glaucoma or a history of ocular surgery were not excluded from the study. The inclusion of such patients may have led to a modest overestimation of the hospital-based frequency of XFS. However, excluding these individuals could also have introduced a different form of selection bias, as XFS is known to be strongly associated with glaucoma and is frequently encountered among patients undergoing cataract evaluation or surgery [5,33]. Therefore, including these patients may better reflect the real-world clinical distribution of XFS in routine ophthalmic practice. Nevertheless, the potential influence of this factor on the estimated hospital-based frequency should be considered when interpreting the results.

## 5. Conclusions

This study provides detailed hospital-based surveillance data of XFS among patients presenting to a regional referral ophthalmology outpatient clinic serving the Northeastern Black Sea coast of Turkey. While these findings should not be extrapolated to population-level estimates, they offer clinically relevant insight into the burden, demographic distribution, and ocular associations of XFS in a regional care-seeking population. From a healthcare perspective, these findings may support regional service planning by underscoring the value of routine XFS screening in older clinic attendees and the need for appropriate resource allocation for glaucoma surveillance and cataract surgery in regional ophthalmology clinics.

In addition, recent global climate and environmental changes have increasingly raised discussions regarding their potential influence on the geographic distribution and frequency of XFS [5,22]. Therefore, comparisons of frequency or prevalence studies conducted over time, from earlier reports to more recent investigations, together with epidemiological research that incorporates environmental factors, may help to better understand the potential contribution of environmental influences to the disease.

## Figures and Tables

**Table 1:** Distribution of gender and age groups of participants with and without XFS diagnosis who presented to the ophthalmology outpatient clinic within a 12-Month Period.

	XFS+		XFS−	
	n	% *	n	% *
Gender				
Male	93	27.6	244	72.4
Female	102	17.0	499	83.0
Chi-square: 14.80; *p*: 0.000				
OR: 1.865; CI: 1.354–2.568				
Age Groups				
40–59 (n: 304)	13	4.3	291	95.7
60–69 (n: 277)	61	22.0	216	78.0
70–79 (n: 257)	87	33.7	171	66.3
80–100 (n: 99)	34	34.3	65	65.7
Total (n: 938)	195	20.8	743	79.2
Chi-square: 87.84; *p*: 0.000				

* Row percentage; CI, 95% confidence intervals; OR, odds ratio.

**Table 2:** The average age distributions by gender of participants with and without XFS diagnosis who presented to the Ophthalmology Outpatient Clinic within a 12-Month period.

	XFS		
Gender (n: 938)	XFS+	XFS−	*p*
	$\;\bar{X}$ ± SE	$\;\bar{X}$ ± SE	
Male (n: 93/244) *	72.20 ± 0.83	65.28 ± 0.70	0.000
Female (n: 102/499)	71.36 ± 0.89	62.26 ± 0.49	0.000
Total (n: 195/743)	71.76 ± 0.61	63.25 ± 0.40	0.000
	*p*: 0.490	*p*: 0.000	

* n: positive/negative.

**Table 3:** The distributions of chronic illnesses of participants with and without XFS diagnosis who presented to the ophthalmology outpatient clinic within a 12-Month period.

	XFS+ (n: 195)		XFS− (n: 743)		
	n	%	n	%	*p*
HT					0.002
yes	112	57.4	339	45.6	
no	83	42.6	404	54.4	
* OR: 1.49; CI: 1.05–2.11; *p*: 0.024					
CAD					0.060
yes	27	13.8	67	9.0	
no	67	86.2	676	91.0	
* OR: 1.17; CI: 0.71–1.95; *p*: 0.359					
DM					1.000
yes	38	19.5	146	19.7	
no	157	80.5	597	80.3	
* OR: 1.11; CI: 0.67–1.54; *p*: 0.951					
HL					0.540
yes	12	6.2	57	7.7	
no	57	93.8	686	92.3	
* OR: 0.94; 0.47–1.86; *p*: 0.853					
CVD					0.160
yes	3	1.5	4	0.5	
no	192	98.5	739	99.5	
* OR: 1.98; 0.42–9.39; *p*: 0.392					
Glaucoma					0.005
yes	46	23.6	111	14.9	
no	149	76.4	632	85.1	
* OR: 2.00; CI: 1.31–3.05; *p*: 0.001					

* Multivariable logistic regression analysis; Adjusted by age and gender odds ratio (OR); 95% confidence intervals (CI).

**Table 4:** The Group Analysis of ophthalmological parameters in patients with and without XFS who presented to the ophthalmology outpatient clinic within a 12-Month Period.

Ophthalmic Parameters	Right Eye			Left Eye		
	XFS+ n: 195 $\;\bar{X}$ ± SE	XFS− n: 743 $\;\bar{X}$ ± SE	*p*	XFS+ n: 195 $\;\bar{X}$ ± SE	XFS− n: 743 $\;\bar{X}$ ± SE	*p*
BCVA (decimal)	0.55 ± 0.25	0.70 ± 0.12	0.000	0.54 ± 0.25	0.68 ± 0.01	0.000
CCT (μm)	533.79 ± 2.61	539.89 ± 1.33	0.040	535.95 ± 2.74	542.97 ± 1.37	0.020
IOP (mmHg)	16.35 ± 0.40	16.12 ± 0.14	0.590	16.19 ± 0.28	16.32 ± 0.14	0.700
c/d	0.21 ± 0.02	0.19 ± 0.01	0.240	0.21 ± 0.02	0.19 ± 0.01	0.160

**Table 5:** The corresponding analysis of ophthalmological parameters in patients with and without XFS who presented to the ophthalmology outpatient clinic within a 12-Month Period.

XFS		Right Eye $\;\bar{X}$ ± SE	Left Eye $\;\bar{X}$ ± SE	*p*
XFS+ (n: 195)				
Bilateral involvement (n: 118)				
	BCVA (decimal)	0.53 ± 0.03	0.53 ± 0.03	0.457
	CCT (μm)	530.69 ± 3.37	532.71 ± 3.50	0.085
	IOP (mmHg)	16.21 ± 0.50	15.95 ± 0.35	0.885
	c/d	0.23 ± 0.02	0.22 ± 0.02	0.312
Unilateral involvement (n: 77)				
Right eye (n: 36)	BCVA (decimal)	0.60 ± 0.06	0.57 ± 0.05	0.285
	CCT (μm)	543.36 ± 6.93	542.50 ± 7.06	0.535
	IOP (mmHg)	18.50 ± 1.26	16.44 ± 0.60	0.032
	c/d	0.19 ± 0.03	0.18 ± 0.03	0.465
Left eye (n: 41)	BCVA (decimal)	0.57 ± 0.52	0.53 ± 0.06	0.126
	CCT (μm)	534.32 ± 4.61	539.51 ± 5.41	0.069
	IOP (mmHg)	14.85 ± 0.40	16.68 ± 0.67	0.004
	c/d	0.15 ± 0.16	0.21 ± 0.32	0.010
XFS− (n: 743)				
	BCVA (decimal)	0.70 ± 0.01	0.68 ± 0.01	0.285
	CCT (μm)	539.89 ± 1.33	542.97 ± 1.37	0.000
	IOP (mmHg)	16.12 ± 0.14	16.32 ± 0.14	0.013
	c/d	0.17 ± 0.01	0.19 ± 0.01	0.996

**Table 6:** The subgroup analysis of ophthalmological parameters in patients with and without XFS who presented to the ophthalmology outpatient clinic within a 12-Month Period.

		Bilateral XFS+ n: 118 $\;\bar{X}$ ± SE	Right XFS+ n: 36 $\;\bar{X}$ ± SE	Left XFS+ n: 41 $\;\bar{X}$ ± SE	XFS− n: 743 $\;\bar{X}$ ± SE	*p*
Right Eye	BCVA (decimal)	0.53 ± 0.03 *	0.60 ± 0.06	0.57 ± 0.52	0.70 ± 0.01 *	0.000
	CCT (μm)	530.69 ± 3.37	543.36 ± 6.93	534.32 ± 4.61	539.89 ± 1.33	0.510
	IOP (mmHg)	16.21 ± 0.50	18.50 ± 1.26 *	14.85 ± 0.40	16.12 ± 0.14	0.002
	c/d	0.23 ± 0.02	0.19 ± 0.03	0.15 ± 0.16	0.17 ± 0.01	0.047
Left Eye	BCVA (decimal)	0.53 ± 0.03	0.57 ± 0.05	0.53 ± 0.06	0.68 ± 0.01 *	0.000
	CCT (μm)	532.71 ± 3.50	542.50 ± 7.06	539.51 ± 5.41	542.97 ± 1.37	0.510
	IOP (mmHg)	15.95 ± 0.35	16.44 ± 0.60	16.68 ± 0.67	16.32 ± 0.14	0.690
	c/d	0.22 ± 0.02	0.18 ± 0.03	0.21 ± 0.32	0.19 ± 0.01	0.250

* Statistically different groups.

## Data Availability

The data that support the findings of this study are not publicly available due to ethical and institutional restrictions, but may be made available from the corresponding author upon reasonable request.
